# A study of the relationship between cytokine levels and the response to anti-VEGF therapy in polypoid choroidal vasculopathy with different choroidal thicknesses

**DOI:** 10.3389/fendo.2023.1307337

**Published:** 2024-01-03

**Authors:** Su Dong, Pan Fan, Haotian Yu, Bo Jiang, Dawei Sun

**Affiliations:** Department of Ophthalmology, The Second Affiliated Hospital of Harbin Medical University, Harbin, China

**Keywords:** biomarker, polypoidal choroidal vasculopathy, anti-vascular endothelial growth factor, cytokine, choroidal thicknesses, pachychoroid

## Abstract

**Purpose:**

Polypoidal choroidal vasculopathy (PCV) is an irreversible retinal choroidal disease. Individuals with PCV exhibit diverse baseline characteristics, including systemic characteristics, ocular traits, metabolic factor levels, and different responses to intravitreal anti-VEGF therapy. This study aims to investigate the pathogenesis of PCV by analyzing the systemic characteristics, ocular traits, and cytokine levels at baseline within a cohort of patients who exhibit different responses to anti-VEGF treatment.

**Methods:**

We conducted a retrospective analysis involving 80 eyes diagnosed with PCV. Patients were categorized into two groups based on responses to suboptimal intravitreal ranibizumab injection therapy: those with suboptimal responses and optimal responses. Aqueous humor samples were collected from the experimental eyes, and cytokine expression levels were assessed using cytometric bead array analysis. All subjects were further stratified into two groups according to the median choroidal thickness. Subsequently, logistic regression analysis and the ROC curve were employed to examine the relationship between cytokine expression levels, choroidal thickness, and anti-VEGF response.

**Results:**

The results revealed that compared to the group of optimal anti-VEGF response, the choroid in the suboptimal response group exhibited a significantly greater thickness. Additionally, compared to the suboptimal anti-VEGF response group, the expression levels of VEGF and VCAM-1 were markedly lower observed in the optimal anti-VEGF response group, while TNF-α showed the opposite trend. Logistic regression analysis indicated that VEGF, VCAM-1, and TNF-α in the aqueous humor were independent risk factors for a suboptimal anti-VEGF response. After adjusting other risk factors, the risk of suboptimal anti-VEGF response decreased to 0.998-fold, 0.997-fold, and 1.294-fold. The AUC values for VEGF, VCAM-1, and TNF-α were determined to be 0.805, 0.846, and 0.897, respectively. Furthermore, the risk of VEGF, VCAM-1, and TNF-α were significantly associated with an increased risk of suboptimal anti-VEGF response after correction for risk factors in the thick choroid group.

**Conclusions:**

Our study demonstrated that PCV exhibits systemic and ocular characteristics variations based on different anti-VEGF responses. The levels of cytokines in aqueous humor were found to have a significant correlation with the anti-VEGF response in PCV. VEGF, VCAM-1, and TNF-α are potential targets for assessing treatment response in thick choroidal PCV.

## Introduction

Polypoid choroidal vasculopathy (PCV) is a retinal and choroidal disease characterized by a network of branching choroidal vessels and polypoid focus at the ends of the vessels. It is a major cause of vision impairment in middle-aged and older adults worldwide, with a high prevalence in Asia. The likelihood of macular involvement and a suboptimal prognosis for vision is high (1–3). Recently, PCV has imposed a substantial economic and health burden on the public health system.

Recent studies indicate that PCV, akin to neovascular age-related macular degeneration (AMD), is a systemic multifactorial disease. Both PCV and typical neovascular AMD involve choroidal neovascularization with blood leakage and sub-retinal hemorrhage in the macular region (2, 4, 5). However, evidence suggests that PCV patients with diverse baseline characteristics exhibit different responses to anti-vascular endothelial growth factor (VEGF) therapy than AMD (6–8). Eyes with PCV often have a thicker subfoveal choroidal thickness (SFCT) than age-related and typical non-neovascular and neovascular AMD (9–11). PCV is now categorized as a pachychoroid disease (12). Recent research has linked SFCT to PCV response to anti-VEGF, indicating that patients with thicker choroidal membranes and thicker SFCT demonstrate poor response to 3-month injections of anti-VEGF compared to patients with thinner SFCT (13). Therefore, staging PCV by choroidal thickness has been suggested (14, 15). A study on macular neovascularization (MNV) classified MNV based on the presence or absence of drusen and the presence or absence of thick choroidal features, revealing distinct cytokine profiles among different subgroups and emphasizing the significance of the choroid and cytokine assessment (16). These differences in systemic characteristics and treatment response variations suggest potential differences in the pathogenetic mechanisms of PCV with varying choroidal thicknesses. However, studies analyzing risk factors according to different choroidal thickness subtypes are scarce, necessitating further research. Identifying individuals with suboptimal anti-VEGF responses is critical for enhancing treatment efficacy accurately and efficiently.

This study elucidates the relationship between cytokines expression levels at different choroidal thicknesses and the response to anti-VEGF. It analyzes numerous risk factors associated with PCV, exploring differences among subgroups with distinct choroidal thicknesses, including baseline clinical data and aqueous humor cytokine expression levels. Furthermore, the study aims to identify risk factors for PCV, explore potential therapeutic targets, and contribute to a deeper understanding of PCV pathogenesis.

## Methods

### Patients and experiment design

This retrospective study adhered to the principles outlined in the Declaration of Helsinki and received approval from the Ethics Committee at the Second Affiliated Hospital of Harbin Medical University (KY2020-261). A total of 80 PCV patients admitted to the Second Affiliated Hospital of Harbin Medical University between December 2018 and June 2022 were selected for the study. Informed consent was obtained from all subjects before their inclusion.

The inclusion criteria were as follows:

1. Confirmation of the initial diagnosis as PCV without prior treatment.
2. Receipt of intravitreal ranibizumab injection (IRI) monotherapy with a 3 + PRN regimen at our hospital.
3. Completion of one year of follow-up after commencing treatment, with comprehensive medical records.

The exclusion criteria were as follows:

1. Presence of eye diseases other than PCV.
2. History of intraocular surgery.
3. Inability to tolerate ranibizumab.
4. Failure to comply with protocol rules for treatment.
5. Severe systemic diseases, such as tumors, liver, or kidney dysfunction.
6. Incomplete clinical data in patient records.

**Figure 1:**
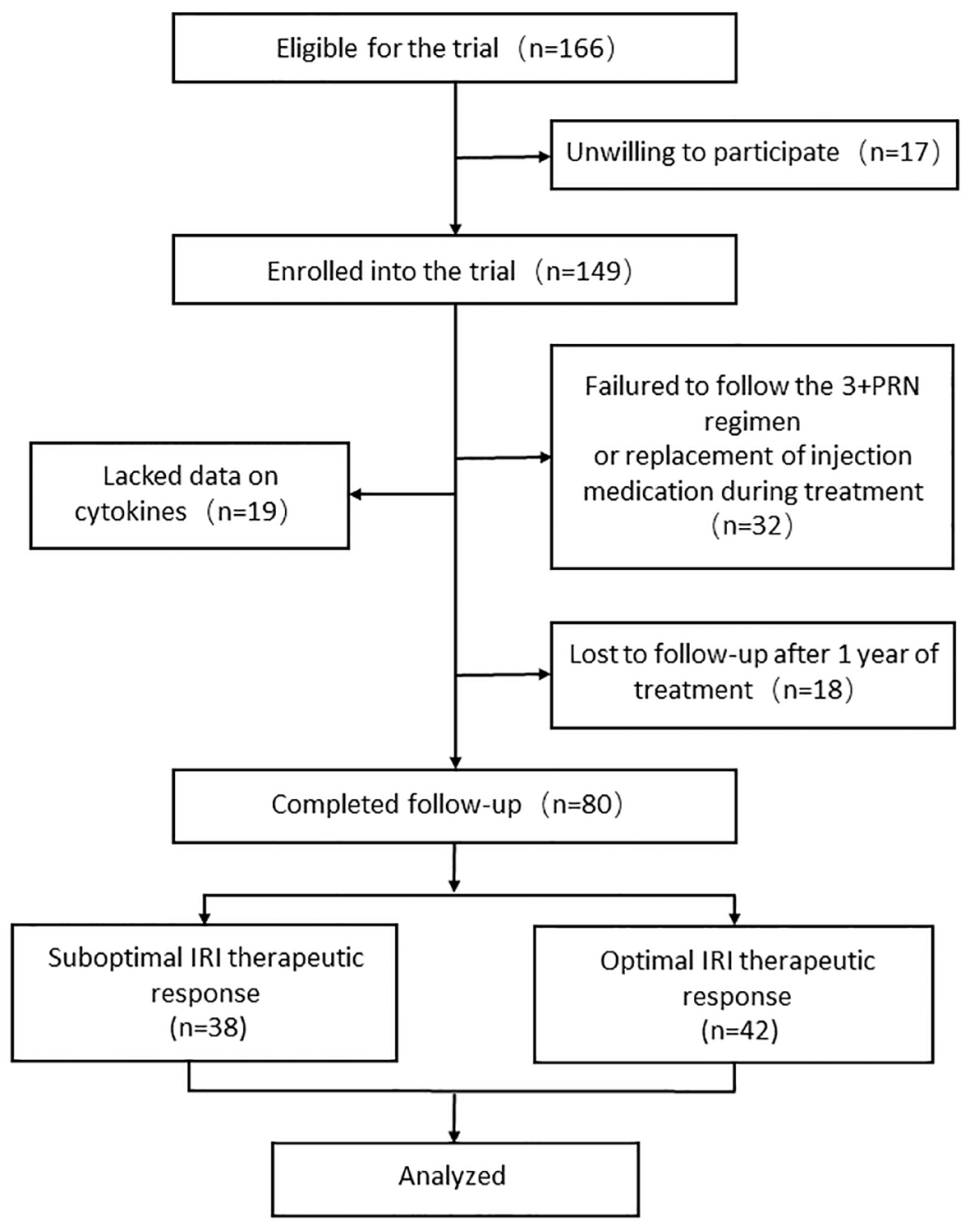
Flow chart of patient recruitment. PRN, pro-re-nata; IRI, intravitreal ranibizumab injection.

“Optimal responders” were characterized as individuals demonstrating the absorption of subretinal fluid and/or intraretinal fluid in the absence of expanding pigment epithelial detachment (PED), or anatomical stabilization (no change in subretinal and/or intraretinal fluid) with an improvement in best-corrected visual acuity (BCVA) of ≥0.1 LogMAR within one year from baseline after initiation of the anti-VEGF treatment. Without meeting these criteria, patients were classified as “suboptimal responders.” The study flow chart is presented in **Figure 1**.

### Data collection and definitions

The data collection and the definition of sociodemographic characteristics (age, gender, height, weight, smoking status, and alcohol consumption), as well as medical history (hypertension, diabetes, systemic diseases, and other prior medical conditions), were derived from hospital medical records.


$$\text{Body mass index }\left(\text{BMI}\right)=\text{weight }\left(\text{kg}\right)/{\text{height}}^{2}\left({\text{m}}^{2}\right)$$

BCVA was recorded using LogMAR measurements. The ocular axis length was measured through an A-ultrasound examination (IOL Master 500, Carl Zeiss, Germany), and the average was recorded after three measurements. Before intravitreal anti-VEGF injections, all patients underwent an optical coherence tomography (OCT) examination (Heidelberg Engineering, Heidelberg, Germany). A volume scan comprising 18 horizontal B-scans covering a 6 × 6 mm area of the macula region centered on the fovea was obtained using SD-OCT. Measurements were taken for the greatest linear dimension (GLD), PED types, and central macular thickness (CMT). SFCT was measured in EDI mode and defined as the thickness from the brush membrane to the choroidal-scleral interface. The PCV patients were categorized into thick and thin choroidal groups based on the median SFCT of all patients: SFCT > 243.0 μm was assigned to the thick choroidal group, while SFCT ≤ 243.0 μm was assigned to the thin choroidal group. Before intravitreal anti-VEGF therapy, 100 μL of aqueous humor was collected from all patients and stored in a refrigerator at –80°C. Cytometric bead arrays were used to test the levels of cytokines.

### Measurement of cytokines in aqueous humor

Following the cytometric bead array (CBA) instructions for experimental operations, which utilized the human VEGF flex set (Bead B8) (No. 558336, BD Bioscience, San Jose, CA, USA); human IL-8 flex set (Bead A9) (No. 558277, BD Bioscience, San Jose, CA, USA); human VCAM-1 flex Set (Bead D6) (No. 560427, BD Bioscience, San Jose, CA, USA); human TNF-α flex set (No. 558273, BD Bioscience, San Jose, CA, USA), for standards and samples were experimented with. For each type of bead, 25 μL of supernatant from centrifuged samples/standard samples was added to the beads working solution (23 μL beads diluent + 0.5 for each type of bead). The mixture was left in darkness for one hour, forming the “beads antibody-antigen PE antibodies” complex. Subsequently, 25 μL of prepared phycoerythrin (PE) working solution (23 μL reagent diluent + 0.5 μL of each type of PE) was added to the tube containing the sample, thoroughly mixed, and left in darkness for two hours. The fluorescence intensity of the complex was tested using flow cytometry devices (FACS Canto TMII, BD Bioscience, San Jose, CA, USA) after washing with wash buffer. Standard curves were plotted using FCAP array v3 (BD Bioscience, San Jose, CA, USA) to calculate the concentration values of each indicator.

### Statistical analysis

Continuous variables are presented as mean ± standard deviation (SD) or median and interquartile spacing (IQR). Categorical variable data are expressed in percentages. The comparison of categorical variables was performed using the χ^2^ test. Continuous variables were compared using the t-test, analysis of variance, Mann-Whitney U test, or Kruskal-Wallis H test, as appropriate. The relationship between aqueous humor cytokine levels and PCV anti-VEGF treatment response was analyzed using logistic regression to calculate the odd ratios (OR) and 95% confidence intervals (CI). Receiver operating characteristic (ROC) curves and the area under the curve (AUC) were employed to determine the sensitivity and specificity of different cytokines expression levels in predicting PCV treatment response. Statistical analysis was conducted using SPSS 26.0 (IBM Corp, New York, NY, USA), and a *P*-value < 0.05 was considered statistically significant.

## Results

### Baseline characteristics according to the response to IRI

This study comprised 80 patients, with an average age of 64 ± 7 years and 53.8% male. Based on the response to anti-VEGF treatment, 47.50% exhibited suboptimal ranibizumab therapeutic response, while 52.50% showed optimal ranibizumab therapeutic response among PCV patients. The baseline characteristics of the suboptimal and optimal ranibizumab therapeutic response groups are detailed in **Table 1**. Compared to the optimal therapeutic response group, patients in the suboptimal therapeutic response group were significantly younger and received more injections (*P* < 0.05). Additionally, the group with optimal therapeutic response had a higher prevalence of hypertension, thinner SFCT, worse BCVA, longer eye axis, greater GLD, and a higher probability of PED occurrence (*P* < 0.05). However, there was no significant difference between the two groups regarding PED typing (*P*>0.05). The group with a suboptimal therapeutic response to anti-VEGF treatment exhibited lower levels of VEGF and VCAM-1 and higher levels of TNF-α in the aqueous humor (*P* < 0.05) (**Table 1**).

**Table 1:** Baseline characteristics according to PCV IRI therapeutic response.

	Total (n=80)	Suboptimal IRI therapeutic response (n=38)	Optimal IRI therapeutic response (n=42)	*P*-value
Age (years)	64 ± 7	61 ± 7	67 ± 6	0.001
Male (n, %)	43(53.8)	22(57.9)	21(50.0)	0.479
Hypertension (n, %)	33(41.3)	11(28.9)	22(52.4)	0.033
Cardiovascular disease (n, %)	20(25.0)	9(23.7)	11(26.2)	0.796
DM (n, %)	15(18.8)	7(18.4)	8(19.0)	0.943
Smoking (n, %)	25(31.3)	11(28.9)	14(33.3)	0.673
Drinking (n, %)	34(42.5)	18(47.4)	16(38.1)	0.402
BMI (kg/m^2^)	26.00(24.97, 26.87)	25.88(24.87, 26.84)	25.98(25.19, 26.96)	0.519
CMT (μm)	374.30(335.63, 416.65)	377.65(329.95, 451.38)	381.75(337.35, 412.25)	0.573
SFCT (μm)	243.00(192.75, 326.75)	303.50(224.00, 373.25)	219.00(176.75, 257.75)	<0.001
BCVA	0.7(0.4, 0.8)	0.5(0.4, 0.7)	0.8(0.5, 0.9)	<0.001
Axial length (mm)	23.59 ± 0.67	23.46 ± 0.60	23.70 ± 0.72	0.104
GLD (μm)	1887.20(1757.35, 2022.13)	1841.55(1761.65, 1942.50)	1948.90(1673.43, 2074.28)	0.098
PED (%)	37(46.25)	13(34.21)	24(57.14)	0.040
Hemorrhagic	14(37.84)	3(23.08)	9(37.50)	
Serous	15(40.54)	6(46.15)	9(37.50)	
Mixed	10(27.03)	4(30.77)	6(25.00)	0.670
Number of injections	8(6, 10)	9(8, 11)	7(5, 9)	0.008
VEGF (pg/mL)	50.55(7.78, 167.53)	10.40(3.35,51.85)	146.00(45.10, 213.73)	<0.001
IL-8 (pg/mL)	38.05 ± 18.75	37.99 ± 17.97	38.10 ± 19.70	0.942
VCAM-1 (pg/mL)	1250.50(618.13, 1669.63)	682.75(503.06,1268.44)	1511.75(1235.30, 2154.30)	<0.001
TNF-α(pg/mL)	7.65(5.65, 14.48)	14.30(10.53, 16.48)	5.80(3.85, 6.95)	<0.001

IRI intravitreal ranibizumab injection; DM diabetes mellitus; BMI body mass index; CMT central macular thickness; SFCT subfoveal choroidal thickness; BCVA the best corrected visual acuity; GLD greatest linear dimension; PED pigment epithelial detachment; VEGF vascular endothelial growth facto; IL-8 Interleukin 8; VCAM-1 vascular cell adhesion molecule-1; TNF-α tumor necrosis factor α.

### Baseline characteristics according to the choroidal thickness

The median SFCT of all patients (243 μm) was taken as the boundary value, dividing the patients into the thick choroid group (48.75%) and the thin choroid group (51.25%). The thin choroidal group exhibited a higher prevalence of hypertensive patients (*P* < 0.05). Compared to the thick choroidal group, the thin choroidal group had a significant worse BCVA, a higher PED case, and fewer injections administered to patients (*P* < 0.05). However, the two groups had no significant difference in PED typing. Concerning cytokines, the thick choroidal group showed lower VEGF and VCAM-1 levels and higher TNF-α levels in aqueous humor (**Table 2**).

**Table 2:** Baseline characteristics according to the choroidal thickness.

	Total (n=80)	Thick group (n=39)	Thin group (n=41)	*P*-value
Age (years)	64 ± 7	60 ± 6	68 ± 6	<0.001
Male (n, %)	40(50.0)	21(53.8)	19(46.3)	0.502
Hypertension (n, %)	33(41.3)	10(25.6)	23(56.1)	0.006
Cardiovascular disease (n, %)	19(23.8)	9(23.1)	10(24.4)	0.890
DM (n, %)	15(18.8)	7(17.9)	8(19.5)	0.858
Smoking (n, %)	25(31.3)	10(25.6)	15(36.6)	0.291
Drinking (n, %)	34(42.5)	18(46.2)	16(39.0)	0.519
BMI (kg/m^2^)	26.00(24.97, 26.87)	25.62(24.73, 26.75)	26.20(25.28, 27.10)	0.124
CMT (μm)	381.28 ± 70.37	387.33 ± 72.79	375.52 ± 68.39	0.457
BCVA	0.7(0.4, 0.8)	0.5(0.4, 0.7)	0.8(0.7, 1.0)	0.004
Axial length (mm)	23.59 ± 0.67	23.30 ± 0.52	23.86 ± 0.68	<0.001
GLD (μm)	1887.00(1757.35, 2002.13)	1763.80(1639.50, 1846.90	1985.80(1917.60, 2075.15)	<0.001
PED (n, %)	37(46.25)	11(28.21)	26(63.41)	0.002
Hemorrhagic	12(32.43)	3(27.27)	9(34.62)	
Serous	15(40.54)	5(45.45)	10(38.46)	
Mixed	10(27.03)	3(27.27)	7(26.92)	0.895
Number of injections	8(6, 10)	9(7, 10)	7(5, 9)	0.004
VEGF (pg/mL)	50.55(7.78, 167.53)	8.90(3.20, 16.20)	166.40(74.50, 245.30)	<0.001
IL-8 (pg/mL)	35.80(24.23, 48.35)	37.00(24.50, 44.00)	35.80(22.50, 51.05)	0.886
VCAM-1 (pg/mL)	1250.50(618.13, 1669.63)	789.25(527.00, 1435.00)	1483.00(894.15, 1768.00)	0.003
TNF-α(pg/mL)	7.65(5.65, 14.48)	12.30(7.10, 15.70)	5.90(3.80, 8.25)	<0.001
Suboptimal IRI therapeutic response (n, %)	38(47.5)	26(66.7)	12(29.3)	0.001

### Relationship between the therapeutic response and various risk factors after IRI treatment for PCV one year

The relationship between the response to anti-VEGF therapy with ranibizumab and various risk factors was analyzed using univariate logistic regression, with the reaction to suboptimal ranibizumab therapy as the dependent variable. The analysis revealed that SFCT, the number of injections, and the expression level of TNF-α were positively associated with a suboptimal ranibizumab treatment response. In contrast, age, hypertension, axial length, PED occurrence, GLD, and the expression levels of VEGF and VCAM-1 were negatively associated with the response to suboptimal ranibizumab treatment (*P* < 0.05) (**Table 3**).

**Table 3:** Relationship between the therapeutic response and various risk factors after IRI treatment for PCV 1 year.

Variables	Suboptimal IRI therapeutic response		
	OR (95%CI)	β	*P*-value
Age (years)	0.891 (0.829-0.957)	-0.116	0.002
Sex			
Male	Reference		
Female	0.727(0.301-1.760)	-0.318	0.480
Hypertension			
No	Reference		
Yes	0.335(0.132-0.847)	-1.094	0.021
Cardiovascular disease			
No	Reference		
Yes	0.875(0.317-2.416)	-0.134	0.796
DM (n, %)			
No	Reference		
Yes	0.960(0.312-2.956)	-0.041	0.943
Smoking (n, %)			
No	Reference		
Yes	0.815(0.315-2.108)	-0.205	0.673
Drinking (n, %)			
No	Reference		
Yes	0.350(0.550-3.312)	0.300	0.512
BMI	0.884(0.588-1.330)	-0.123	0.554
CMT	1.003(0.996-1.009)	0.003	0.394
SFCT	1.013(1.006- 1.020)	0.013	<0.001
BCVA	0.009(0.001-0.098)	-4.694	<0.001
Axial length	0.562(0.278-1.134)	-0.576	0.108
GLD (μm)	0.998(0.996- 1.000)	-0.002	0.104
PED	0.390(0.157-0.966)	-0.942	0.042
Hemorrhagic	Reference		
Serous	2.000(0.378-10.578)	0.693	0.415
Mixed	2.000(0.324-12.329)	0.693	0.455
Number of injections	1.476(1.183-1.840)	0.389	0.001
VEGF	0.988(0.981-0.994)	-0.012	<0.001
IL-8	1.000(0.976-1.023)	0	0.981
VCAM-1	1.001(0.997-0.999)	-0.002	<0.001
TNF-α	1.352(1.181-1.548)	0.302	<0.001

OR odds ratios, CI confidence interval, β regression coefficient.

### Association between levels of various cytokines in the aqueous humor and IRI response

As presented in **Table 4**, factors with *P* ≤ 0.1 were incorporated into the multivariate logistic regression analysis model to analyze the correlation between aqueous humor cytokine levels and the response to ranibizumab anti-VEGF therapy. The suboptimal ranibizumab treatment served as the dependent variable, and there was no evidence of multicollinearity among the independent variables. Confounding factors were not adjusted in Model 1. Model 2 was adjusted for systematic factors such as age, sex, and hypertension. Model 3 was adjusted for sex, age, hypertension, choroidal thickness classification, ocular axis length, PED, and GLD (BCVA and injection numbers were excluded from the adjustment due to being outcomes of the disease). The analysis revealed that VEGF, VCAM-1, and TNF-α were independent risk factors for the response to suboptimal ranibizumab treatment (*P* < 0.05) (**Table 4**).

**Table 4:** Association between cytokines and IRI therapeutic response of PCV.

Variables	Suboptimal IRI therapeutic response					
	Model 1		Model 2		Model 3	
	OR (95%CI)	*P*-value	OR (95%CI)	*P*-value	OR (95%CI)	*P*-value
VEGF	0.988(0.981-0.994)	<0.001	0.991(0.984-0.997)	0.007	0.998(0.979-0.998)	0.013
VCAM-1	1.001(0.997-0.999)	<0.001	0.998(0.997-0.999)	<0.001	0.997(0.996-0.999)	<0.001
TNF-α	1.352(1.181-1.548)	<0.001	1.304(1.133-1.500)	<0.001	1.294(1.118-1.497)	0.001

Model 1: unadjusted.

Model 2: adjusted for gender, age and hypertension;

Model 3: adjusted for gender, age, hypertension, choroidal thickness classification, axial length, PED presence and GLD (P ≤ 0.1 included in multivariate logistic regression analysis).

### The predictive value of aqueous humor cytokine levels for the IRI response

**Figure 2:**
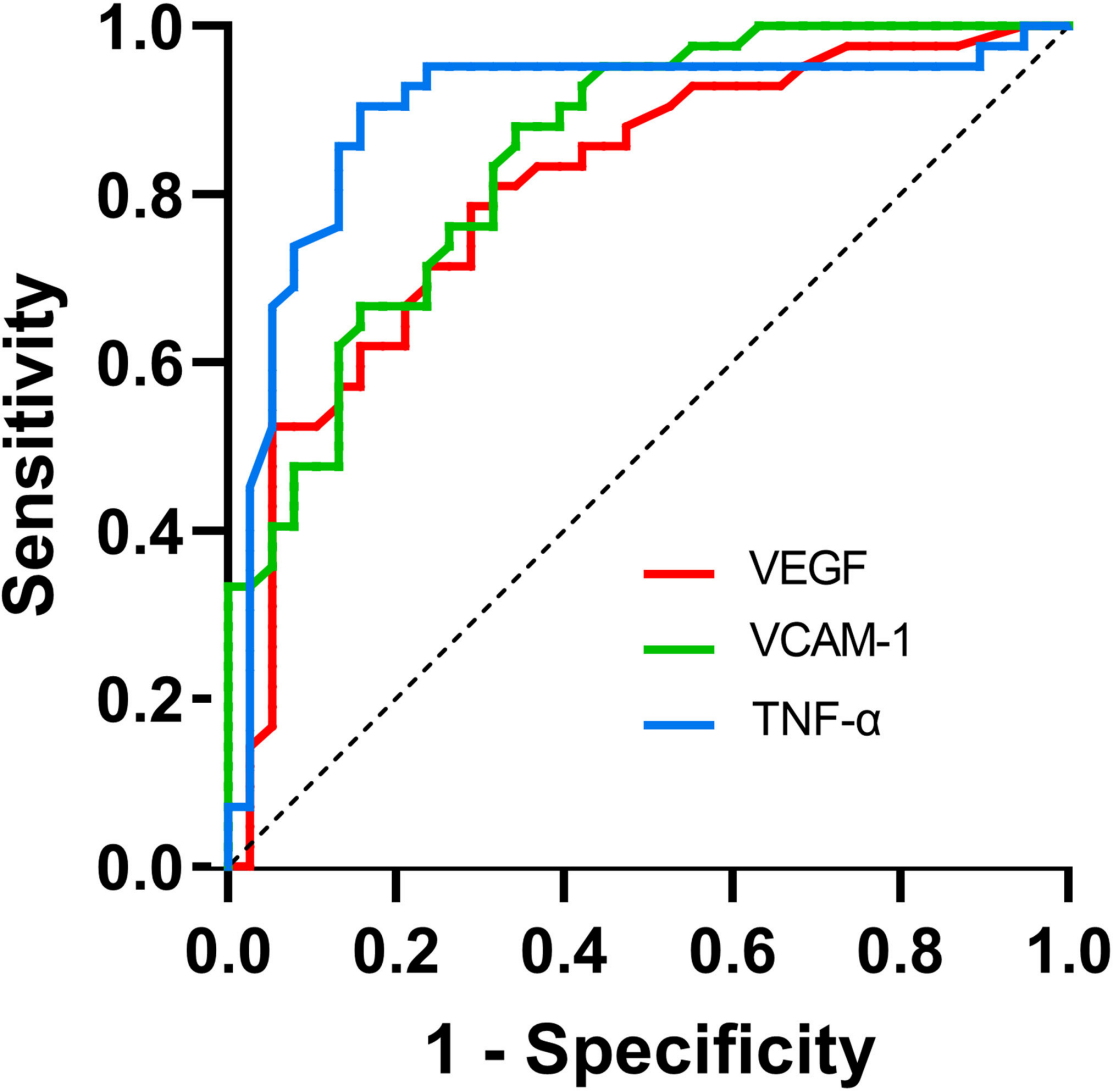
ROC curve for the use of cytokines in the detection of suboptimal IRI therapeutic response.

The ROC curves illustrating suboptimal ranibizumab therapeutic response and aqueous humor cytokines levels are depicted in **Figure 2**. The AUC of suboptimal anti-VEGF treatment response assessed by VEGF was 0.805 (95% CI 0.707-0.902), yielding an optimal cut-off value of 43.900 at a sensitivity of 71.1% and a specificity of 78.6% (*P*<0.05). VCAM-1 demonstrated an AUC of 0.846 (95% CI: 0.763–0.929) and an optimal cut-off value, sensitivity, and specificity of 862.00, 65.8%, and 88.1%, respectively (*P* < 0.05). The AUC assessed by TNF-α was 0.897 (95% CI: 0.818–0.976), with the optimal cut-off value, sensitivity, and specificity being 8.450%, 84.2%, and 90.5%, respectively (*P* < 0.05) (**Table 5**).

**Table 5:** The predictive value of cytokines for therapeutic response of PCV.

Variables	AUC	95%CI	Cut-off value	Sensitivity (%)	Specificity (%)
VEGF	0.805	0.707-0.902	43.900	71.1	78.6
VCAM-1	0.846	0.763-0.929	862.00	65.8	88.1
TNF-α	0.897	0.818-0.976	8.450	84.2	90.5

AUC area under the curve.

### Correlations between cytokines and treatment response in different choroidal thickness groups

For further investigation, we included cytokines (VEGF, VCAM-1, and TNF-α) that exhibited differential expression in the two groups. Patients were categorized into thin and thick choroidal groups based on the median SFCT. Logistic regression analysis was conducted on both groups, with the response to suboptimal ranibizumab treatment as the dependent variable. In both the thick and thin choroidal groups, we observed that VEGF, VCAM-1, and TNF-α were identified as risk factors for suboptimal ranibizumab therapeutic response. After adjusting for all confounding risk factors (Model 5, Model 6), VEGF, VCAM-1, and TNF-α remained independent risk factors for obtaining a suboptimal ranibizumab treatment response in the thick choroid group. Only VCAM-1 emerged as an independent risk factor for obtaining a suboptimal ranibizumab treatment response in the thin choroid group (**Table 6**).

**Table 6:** Correlations between cytokines and IRI therapeutic response in different choroidal thickness groups.

Disease types	Variables	Suboptimal IRI therapeutic response					
		Model 4		Model 5		Model 6	
		OR (95%CI)	*P*-value	OR (95%CI)	*P*-value	OR (95%CI)	*P*-value
Thick group	VEGF	0.946(0.903-0.991)	0.020	0.944(0.896-0.995)	0.032	0.967(0.937-0.998)	0.037
	VCAM-1	0.998(0.997-0.999)	0. 003	0.998(0.997-0.999)	0.004	0.998(0.996-0.999)	0.005
	TNF-α	1.406(1.122-1.761)	0.003	1.397(1.009-1.777)	0.006	1.394(1.084-1.792)	0.010
Thin group	VEGF	0.990(0.981-0.999)	0.028	0.992(0.983-1.001)	0.077	0.992(0.983-1.001)	0.094
	VCAM-1	0.998(0.996-0.999)	0.010	0.997(0.995-1.000)	0.029	0.997(0.991-1.003)	0.028
	TNF-α	1.241(1.048-1.470)	0.013	1.189(0.995-1.421)	0.057	1.149(0.956-1.382)	0.139

Model 1: unadjusted;

Model 2: adjusted for gender, age and hypertension;

Model 3: adjusted for gender, age, hypertension, axial length, PED suffered and GLD.

## Discussion

Our study demonstrated a significant correlation between systemic characteristics and cytokine expression levels in the aqueous humor at baseline with the anti-VEGF treatment response in patients with PCV. The baseline characteristics of PCV varied among subgroups categorized by different choroidal thicknesses. Our study is novel in exploring baseline clinical characteristics in the same cohort of patients grouped according to both parameters.

In this study, we observed that patients with a suboptimal anti-VEGF response had a thicker choroid than those with an optimal anti-VEGF response. Upon observing baseline features, we identified numerous systemic and ocular characteristics similarities between the group with a suboptimal IRI response and the group with a thicker choroid. There were even more similarities between the group with optimal IRI response and the group with a thinner choroid. The group with optimal IRI response and the group with a thin choroid were characterized by older patients and a higher prevalence of hypertension. Regarding ocular characteristics, both groups exhibited worse visual acuity, a higher probability of suffering from PED, a larger GLD, a longer ocular axis length, and fewer injections within a year. The observation of optimal IRI response may result from a floor effect of worse visual acuity, signifying a significant treatment effect. Besides, fewer injections also suggest a favorable responsiveness to anti-VEGF in this patient group. Our aqueous humor cytokine assay in PCV subject eyes revealed higher VEGF and VCAM-1 expression levels in the group with optimal anti-VEGF response and group with a thinner choroid. In contrast, TNF-α expression was lower in the both groups.

Currently, the classification of PCV remains controversial, and whether PCV is considered one of the subtypes of AMD or a separate clinical disease requires further research, notably (10). PCV is presently categorized as a pachychoroid disease, sharing standard features such as a reduced volume of the capillary and Sattler layers, dilation of the large vessel layer, and secondary retinal pigment epithelial dysfunction or neovascularization (12, 17, 18). Not all patients diagnosed with PCV exhibit thick choroid, and our study confirms that PCV with different choroidal thicknesses presents different ocular characteristics (19–21). From a genetic perspective, thick choroid and choroidal vascular hyperpermeability are important features of pachychoroid spectrum diseases. Previous studies have suggested the classification of PCV into two subtypes: pachychoroid neovasculopathy (PNV) and drusen-driven PCV. Examining PCV from a genetic viewpoint may assist in reorganizing the subtypes of drusen-driven AMD and pachychoroid spectrum diseases (22). Recently, the literature has further explored the distribution of choroidal thickness, identifying baseline nasal peripapillary choroidal thickness (nPCT) and SFCT/nPCT ratios as useful as biomarkers reflecting clinical outcomes after anti-VEGF therapy for PCV (23). Our studies revealed that patients with thinner choroidal thickness have a higher incidence of chronic hypertension, a viewpoint supported by numerous studies. Chronic hypertension and heart failure may reduce choroidal thickness, as indicated by a review and meta-analysis examining the relationship between choroidal thickness and cardiovascular disease. Both coronary artery disease and carotid artery stenosis could be linked to the pathology, resulting in decreased blood flow and choroidal thickness. Conversely, acute hypertension increases choroidal thickness, possibly due to the inability of choroidal vasoregulation to withstand acute hypertension-induced choroidal ischemia, leading to altered choroidal permeability and interstitial fluid accumulation, thereby increasing SFCT (24).

Previous genetic studies have identified several causative factors associated with neovascular AMD (nAMD), including VEGF, MCP-1, soluble intercellular adhesion molecule-1, soluble vascular cell adhesion molecule-1, IL-6, IL-8, IL-10, C-reactive protein, human growth factor, tumor necrosis factor-α, IL-31, leukemia inhibitory factor, and stromal cell-derived factor-1-α (25–27). Literature has compared the phenotypic/genetic differences between PNV and nAMD, revealing distinct genetic risk scores calculated from AMD susceptibility genes and different etiologies for the two diseases (28). Recent literature has discussed and compared inflammatory cytokine profiles between nAMD and PNV. The study found that VEGF-A was significantly lower in the PNV group than in the nAMD group but almost identical to the control group, affirming differences in cytokine expression between the two diseases (29, 30). IL-8, a chemokine that promotes vascular endothelial proliferation (31–33), has been found to have higher levels in patients with PCV or nAMD in aqueous humor (29, 30). Similarly, our study observed higher overall expression of IL-8 in PCV. However, there was no difference in IL-8 in either subgroup, indicating a minimal correlation between IL-8 and the degree of anti-VEGF response and choroidal thickness. Vitreous anti-VEGF therapy is widely used to treat PCV, helping eliminate retinal fluid and improve BCVA in the short term (34–36). While anti-VEGF is one of the most effective therapies for secondary CNV, the literature reports lower VEGF concentrations in aqueous humor in patients with PNV than those with drusen-associated MNV, and the efficacy of anti-VEGF therapy for PCV is not conclusive (29). Consequently, we hypothesize that thinner choroidal PCV subtypes share similar mechanisms to nAMD regarding cytokine pathogenesis, and elevated VEGF may be reciprocally associated with choroidal ischemia and atrophy of the inner vascular layer. Thick choroidal PCV may have independent pathogenesis and therapeutic targets compared to thin choroidal PCV and nAMD. Improved visual acuity after anti-VEGF therapy has been associated with lower serum levels of TNF-α, and targeting TNF-α may facilitate the treatment of neovascular AMD (37). In our study, TNF-α expression was higher in the thick choroid group and was identified as an independent risk factor for anti-VEGF therapy. Therefore, combined inhibition of VEGF and TNF-α may be a potential option for treating PCV. Our study confirms that low VEGF, VCAM-1, and high expression levels of TNF-α are independent risk factors for suboptimal anti-VEGF response, especially in the thick choroidal group. It demonstrates the relationship between VEGF, VCAM-1 and TNF-α and the anti-VEGF response in PCV with different choroidal thicknesses, providing evidence not documented to date. These results contribute to an improved understanding of anti-VEGF therapy of PCV.

In the future, further research is needed to comprehend the distinctions between typical AMD and PCV and to unravel how and why they differ. Additionally, we need to understand how the immune system in the choroid changes with age and disease. Specific focus should be directed towards the molecular basis of PCV, including a comprehensive understanding of the functionality of genes associated with PCV risk, with a particular emphasis on the choroid. In future clinical practice, evaluating ocular conditions, such as aqueous humor cytokine testing in patients with thick choroidal PCV requiring anti-VEGF therapy, can be instrumental in determining treatment response. This approach can facilitate more timely, accurate, and appropriate treatment based on the obtained results. Moreover, our study contributes valuable insights that can guide strategies for discovering new therapeutic targets for PCV.

## Limitations

Our study provides a foothold in predicting baseline characteristics and diagnosing the anti-VEGF response in PCV with different choroidal thicknesses. However, several limitations should be considered. The study population was derived from a single center, and the sample size was small. Due to the lack of automated choroidal thickness measurement software in current OCT equipment, manual assessment was performed on all SD-OCT images, and repeated measurements were conducted to mitigate potential observational errors associated with manual measurements. Normal subjects and patients with AMD were not included as controls in this experiment, and we solely explored the conditions of patients with PCV of different subtypes. Therefore, the generalizability of our results could be improved, and further studies are needed to validate our findings.

## Conclusions

In summary, our study reveals that PCV with different anti-VEGF responses differ in systemic and ocular characteristics. The cytokine levels in the aqueous humor significantly correlate with the anti-VEGF response in PCV. Specifically, VEGF, VCAM-1, and TNF-α are potential targets for assessing treatment response in thick choroidal PCV. In the future, aqueous humor cytokine testing and choroidal thickness assessment hold promise for risk management in PCV patients requiring anti-VEGF therapy.

## Data availability statement

The original contributions presented in the study are included in the article/supplementary material. Further inquiries can be directed to the corresponding authors.

## Ethics statement

The studies involving humans were approved by the Ethics Review Committee of the Second Affiliated Hospital of Harbin Medical University. The studies were conducted in accordance with the local legislation and institutional requirements. The participants provided their written informed consent to participate in this study.

## Author contributions

SD: Data curation, Formal Analysis, Investigation, Methodology, Writing – original draft. PF: Data curation, Formal Analysis, Writing – original draft. HY: Data curation, Formal Analysis, Writing – original draft. BJ: Conceptualization, Writing – review & editing, Supervision. DS: Conceptualization, Writing – review & editing, Supervision.
